# Characterization of Melanogenesis Inhibitory Constituents of *Morus alba* Leaves and Optimization of Extraction Conditions Using Response Surface Methodology

**DOI:** 10.3390/molecules20058730

**Published:** 2015-05-14

**Authors:** Ji Yeon Jeong, Qing Liu, Seon Beom Kim, Yang Hee Jo, Eun Jin Mo, Hyo Hee Yang, Dae Hye Song, Bang Yeon Hwang, Mi Kyeong Lee

**Affiliations:** College of Pharmacy, Chungbuk National University, Cheongju, Chungbuk 362-763, Korea; E-Mails: opsl7945@naver.com (J.Y.J.); liuqing7115@hotmail.com (Q.L.); suntiger85@hanmail.net (S.B.K.); qow0125@naver.com (Y.H.J.); mej2403@nate.com (E.J.M.);tmdgy1203@hanmail.net (H.H.Y.); thdek90@naver.com (D.H.S.); byhwang@chungbuk.ac.kr (B.Y.H.)

**Keywords:** *Morus alba*, melanogenesis, optimization, tyrosinase, melanin, phenolic content

## Abstract

Melanin is a natural pigment that plays an important role in the protection of skin, however, hyperpigmentation cause by excessive levels of melatonin is associated with several problems. Therefore, melanogenesis inhibitory natural products have been developed by the cosmetic industry as skin medications. The leaves of *Morus alba* (Moraceae) have been reported to inhibit melanogenesis, therefore, characterization of the melanogenesis inhibitory constituents of *M. alba* leaves was attempted in this study. Twenty compounds including eight benzofurans, 10 flavonoids, one stilbenoid and one chalcone were isolated from *M. alba* leaves and these phenolic constituents were shown to significantly inhibit tyrosinase activity and melanin content in B6F10 melanoma cells. To maximize the melanogenesis inhibitory activity and active phenolic contents, optimized *M. alba* leave extraction conditions were predicted using response surface methodology as a methanol concentration of 85.2%; an extraction temperature of 53.2 °C and an extraction time of 2 h. The tyrosinase inhibition and total phenolic content under optimal conditions were found to be 74.8% inhibition and 24.8 μg GAE/mg extract, which were well-matched with the predicted values of 75.0% inhibition and 23.8 μg GAE/mg extract. These results shall provide useful information about melanogenesis inhibitory constituents and optimized extracts from *M. alba* leaves as cosmetic therapeutics to reduce skin hyperpigmentation.

## 1. Introduction

Melanin is a dark macromolecular pigment produced by melanogenesis. It determines skin colors and also plays an important role in protecting the skin from UV radiation and toxic chemicals. However, excessive accumulation of melanin in specific parts induces diverse pigmentation problems [1,2]. Melanogenesis is a complex biosynthetic process controlled by a cascade of enzymatic reactions. Tyrosinase, the enzyme that catalyzes the initial step of melanin synthesis, is the rate-limiting enzyme of melanogenesis. Melanin synthesis are also regulated by various melanogenic enzymes such as the tyrosinase-related protein 1 (TRP-1) and TRP-2, and cellular signaling [3,4]. Melanogenesis inhibitors have become important targets, especially for cosmetic products to treat hyperpigmentation [5,6,7,8]. 

*Morus alba* is a deciduous tree that belongs to the Moraceae family. This tree is widely distributed in Asia and all parts of this tree including roots, fruits, twigs and leaves are of great importance in traditional medicine. Among them, the leaves of *M. alba* has been used in traditional medicine for the treatment of metabolic disorders [9]. Recently, the anti-melanogenesis activity of extracts from *M. alba* leaves also have been reported [10,11]. In addition, stilbenoids and chalcones of *M. alba* leaves were reported as active constituents that inhibit tyrosinase activity and reduce melanin content [12,13,14,15]. Therefore, *M. alba* extracts and their constituents are suggested as promising natural sources for dietary supplements or for development as cosmetic products, especially for whitening.

For the development of products using plants, an efficient extraction procedure is indispensable. During the extraction procedure, many factors such as extraction solvent, extraction time, extraction temperature and solid-liquid ratios affect the composition of the resulting extract as well as its biological activity [16,17,18]. Therefore, optimization of extraction condition is essential for maximum efficacy. Response surface methodology that consists of mathematical and statistical techniques is an efficient tool for optimization. Response surface methodology can take into several factors simultaneously, thus it is fast and reasonable for optimization of extraction conditions, especially in the case of several variables [19,20,21].

In the present study, we attempted to characterize the melanogenesis inhibitory constituents of *M. alba* leaves. For optimization, response surface methodology with a three-level-three-factor Box-Behnken design (BBD) was employed to evaluate the effect of multiple factors of the extraction conditions such as methanol concentration, extraction time and extraction temperature on tyrosinase activity and total phenolic content.

## 2. Results and Discussion

### 2.1. Characterization of Compounds 

**Figure 1:**
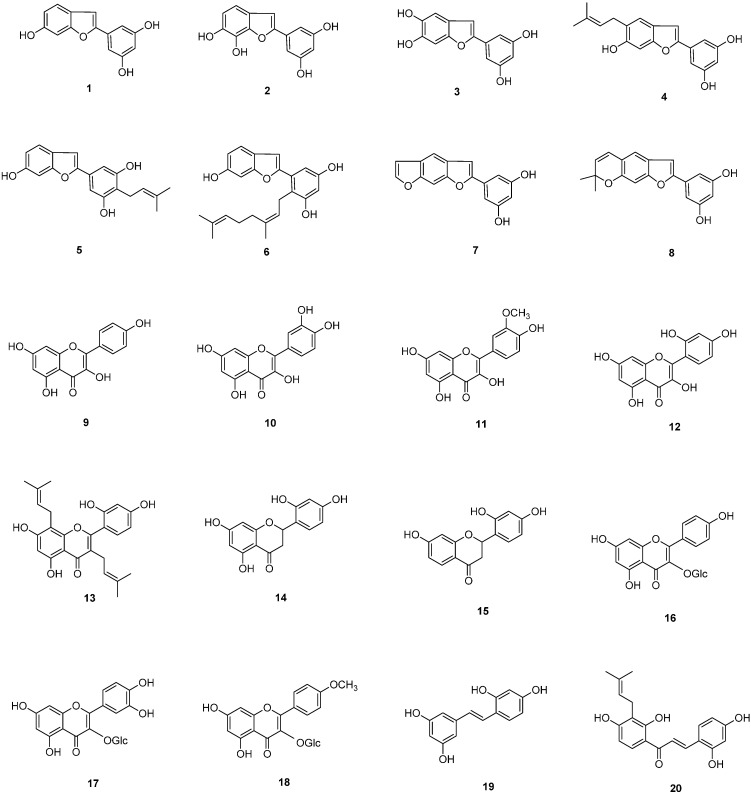
Chemical structures of compounds **1**–**20**.

The leaves of *M. alba* were extracted twice with 80% MeOH, which yielded the methanolic extract. The methanolic extract was then fractionated into *n*-hexane, CH_2_Cl_2_, EtOAc and *n*-BuOH fractions. Further fractionation of the CH_2_Cl_2_ and EtOAc-soluble fractions resulted in the isolation of 20 compounds (Figure 1). 

The structures of the isolated compounds were determined as eight 2-phenylbenzofurans, moracin M (**1**), 2-(3,5-dihydroxyphenyl)-5,6-dihydroxybenzofuran (**2**), wittifuran E (**3**), moracin N (**4**), moracin C (**5**), albafuran A (**6**), moracin X (**7**), and morunigrol C (**8**); ten flavonoids: norartocarpetin (**9**), kaempferol (**10**), quercetin (**11**), isorhamnetin (**12**), kuwanon C (**13**), steppogenin (**14**), 7,2',4'-trihydroxyflavanone (**15**), astragalin (**16**), quercetin-3-*O*-β-d-glucopyranoside (**17**), and kaempferide 3-*O*-β-d-glucoside (**18**), one stilbenoid: oxyresveratrol (**19**), and one chalcone: morachalcone A (**20**) by spectroscopic analysis and comparison of literature values [14,22,23,24,25,26,27,28,29,30,31,32,33].

### 2.2. Effect on Melanogenesis

**Figure 2:**
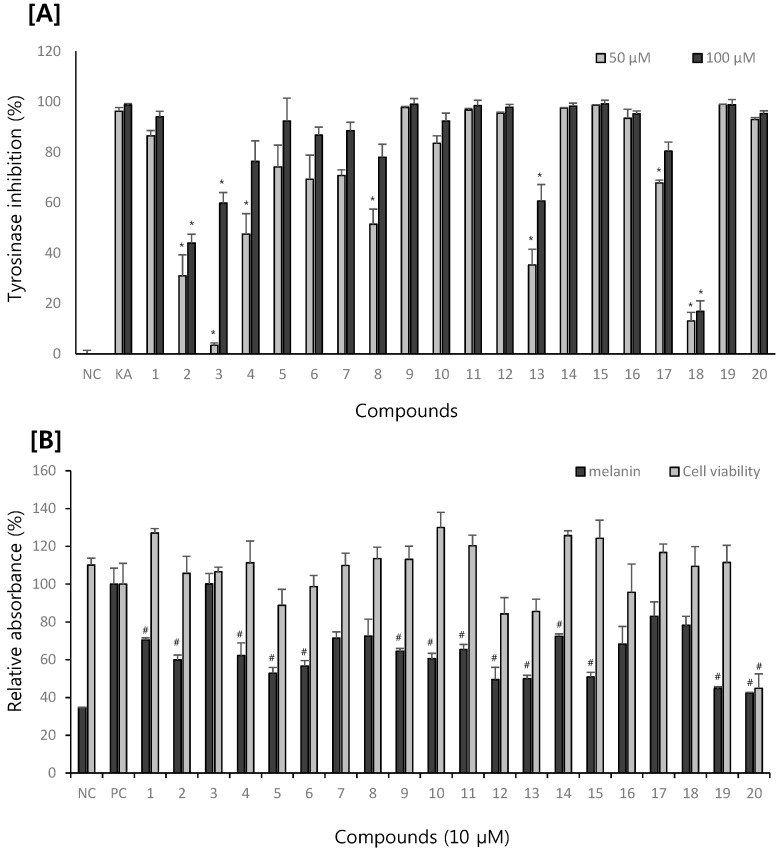
Effects of compounds **1**–**20** on (**A**) tyrosinase inhibition and (**B**) melanin content in B16F10 melanoma cells. Data was expressed as mean ± S.D. (n = 3). NC, normal control; PC, α-MSH stimulated positive control; KA, kojic acid. * *p* < 0.05 compared to NC (A), # *p* < 0.05 compared to PC (B).

The effect of the isolated compounds on melanogenesis was first evaluated *in vitro* using mushroom tyrosinase. Among the compounds isolated from *M. alba* leaves, compound **18** showed the most potent inhibition on tyrosinase activity, followed by compounds **2**, **3**, **4**, **8**, **13** and **17** (Figure 2A). The melanogenesis inhibitory effect of isolated compounds was also evaluated by measuring the melanin content in B16F10 melanoma cells. Stimulation of B16F10 melanoma cells with α–MSH significantly increased the melanin synthesis. However, all the compounds except for compounds **3** and **20** reduced the melanin content at 10 μM without any cytotoxicity (Figure 2B). Taken together, phenolic compounds of *M. alba* leaves are active constituents for anti-melanogenesis activity. 

### 2.3. Optimization of Extraction Conditionsadmas

#### 2.3.1. Extraction Method Development

The optimized extraction conditions for maximum extraction efficacy was investigated using response surface methodology. In our present study, phenolic compounds significantly inhibited melanogenesis, therefore, tyrosinase inhibition and phenolic content were chosen as target responses. Three extraction variables, namely extraction solvent, extraction temperature and extraction time were selected on the basis of preliminary single factor experiments. Variable ranges were set as *X_1_* (extraction solvent): methanol concentration 0%–100%; *X_2_* (extraction temperature): 20–60 °C and *X_3_* (extraction time: 2–24 h. To evaluate any multiple effects of the extraction factors on tyrosinase inhibition and phenolic content, a three-level-three-factors Box-Behnken design (BBD) was employed, as shown in Table 1.

**Table 1:** A Box-Behnken design for independent variables and their responses.

Run	Actual Variables			Observed Values	
	MeOH Concentration (%)	Extraction Temperature (°C)	Extraction Time (h)	Tyrosinase Inhibition (%)	Total Phenolic Content (μg GAE/mg extract)
1	0	60	13	0.0	4.1
2	0	20	13	0.0	5.0
3	50	40	13	30.9	20.4
4	50	20	2	20.0	25.3
5	0	40	2	0.0	13.9
6	100	40	24	83.1	20.1
7	50	20	24	15.3	25.1
8	0	40	24	0.0	16.7
9	50	40	13	22.0	21.7
10	100	40	2	81.6	15.7
11	100	60	13	81.5	16.8
12	50	60	2	46.0	25.6
13	50	40	13	59.8	20.4
14	100	20	13	82.5	7.6
15	50	60	24	36.4	23.9

The significance of each coefficient was determined using *t*-test and *p*-values. Multiple regression analysis on the experiment data yielded the second-order polynomial regression equations as follows:

Tyrosinase inhibition = 37.86 + 60.40*X*_1_ (1)

(2) $$\text{Phenolic content}=21.34+3.19X_{1}+1.25X_{2}\;–10.41X_{1}^{2}\;–2.48X_{2}^{2}+5.36X_{3}^{2}+2.63X_{1}X_{2}$$

For the determination of significance and suitability of regression equation, ANOVA analysis was used. Greater *F*-value and smaller *p*-value were considered as significant. Lack of fit was also determined to check the quality of the model. ANOVA analysis of the models for tyrosinase inhibition and total phenolic content showed the high *F*-values (16.83 and 44.00, respectively), low *p*-values (0.003 and 0.000, respectively) and insignificant *p*-value (0.897 and 0.247, respectively) of lack of fit, which supported the reliability of this model (Table 2).

**Table 2:** ANOVA analysis for second order polynomial models for tyrosinase inhibition and total phenolic content.

[A] Tyrosinase inhibition					
**Variation**	**Sum of Square**	**Degree of Freedom**	**Mean Square**	***F*-Value**	***p*-Value**
Model	30301.50	9	3366.83	16.83	0.003
Residual error	1000.20	5	200.04		
Lack-of-fit	219.40	3	73.30	0.19	0.897
Pure error	11.25	2	5.62		
Total	31301.70	14			
R^2^ = 0.968, adjusted R^2^ = 0.911					
[B] Total phenolic content					
**Variation**	**Sum of Square**	**Degree of Freedom**	**Mean Square**	***F*-Value**	***P*-Value**
Model	687.36	9	76.37	44.00	<0.001
Residual error	8.68	5	1.74		
Lack-of-fit	7.18	3	2.39	3.19	0.247
Pure error	1.50	2	0.75		
Total	696.04	14			
R^2^ = 0.987, adjusted R^2^ = 0.965					

**Figure 3:**
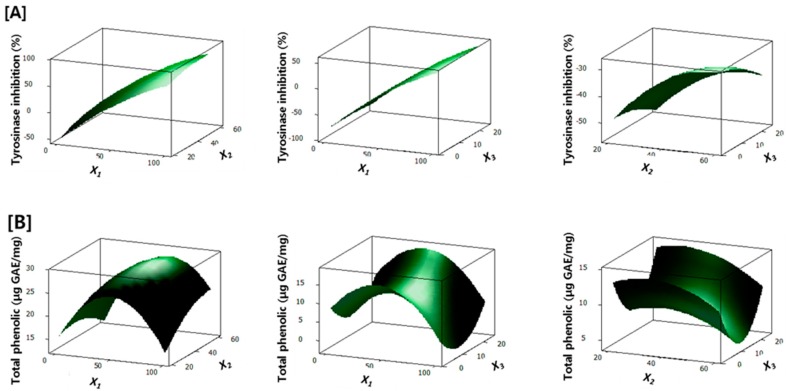
Response surface plots show the effect of extraction variables on (**A**) tyrosinase inhibition and (**B**) total phenolic content. Three variables are methanol concentration (*X_1_*), extraction temperature (*X_2_*) and extraction time (*X_3_*).

Three dimensional response surface plots for tyrosinase inhibition and total phenolic content are shown in Figure 3. These response surface plots clearly showed linear effect of methanol concentration on tyrosinase inhibition. 

Tyrosinase inhibition was greatly increased with an increase in methanol concentration ($X_{1}$), whereas extraction temperature ($X_{2}$) and extraction time ($X_{3}$) showed only slight quadratic effects on tyrosinase inhibition. Three dimensional response surface plots for total phenolic content, however, showed the quadratic effect of methanol concentration, extraction temperature and extraction time. Total phenolic content increased with increasing methanol concentration and extraction temperature but decreased with continuing increase of methanol concentration and extraction temperature. 

#### 2.3.2. Optimization of Extraction Parameters and Verification

Based on our results, an optimization for extraction condition for both responses was evaluated and verified by experiment. The target was to obtain maximum tyrosinase inhibition and high total phenolic content.

Optimal condition for both maximum tyrosinase inhibition and total phenolic content was determined as methanol concentration of 85.2%; temperature of 53.2 °C; and extraction time of 2.0 h, which predicted 75.0% tyrosinase inhibition and 23.8 μg GAE/mg extract. These conditions gave 74.7% tyrosinase inhibition and 24.7 μg GAE/mg extract, which showed good correlation between predicted and actual values (Table 3). Thus, this model can be used to optimize the *M. alba* leaves extraction process.

**Table 3:** Predicted and observed values of tyrosinase inhibition and total phenolic content under optimized condition.

Extraction Condition			Tyrosinase Inhibition ^a^		Total phenolic Content ^b^	
MeOH Concentration (%)	Extraction Temperature (°C)	Extraction Time (h)	Predicted	Observed	Predicted	Observed
85.2	53.2	2.0	75.0	74.7	23.8	24.7

^a^ Tyrosinase inhibition (%) was measured at 100 μg/mL; ^b^ Total phenolic content was expressed as μg GAE/mg extract.

### 2.4. Discussion

In our present study, twenty compounds were isolated from *M. alba* leaves and their effects on melanogenesis was evaluated by direct measuring the inhibitory effect on tyrosinase activity and melanin content in B16F10 melanoma cells. The inhibitory effect of isolated compounds on tyrisonase activity was first assessed *in vitro* using mushroom tyrosinase. Tyrosinase catalyzes the first rate-limiting step in the melanogenesis and plays a pivotal role in melanin synthesis [34,35]. In our present study, compound **18** showed the most potent inhibition on tyrosinase activity, followed by compounds **2**, **3**, **4**, **8**, **13** and **17**. Considering the structure of compounds **1**–**20**, all the compounds isolated from *M. alba* leaves are phenolic compounds and can be divided into benzofurans **1**–**8**, flavonoids **9**–**18**, a stilbenoid **19** and a chalcone **20**. Concerning the structure activity relationship, flavonoids **9**–**18** exerted strong inhibition at 50 μM and 100 μM. However, addition of prenyl groups (compound **13**) or replacement of the 4ʹ-hydroxyl group by a methoxyl moiety (compound **18**) reduced the inhibitory activity. 2-Phenylbenzofurans are also good inhibitors in our study. Moracin M (**1**), which is 2-(3,5-dihydroxyphenyl)-5-hydroxybenzofuran is most potent and addition of hydroxyl or prenyl groups to the benzofuran skeleton (compounds **2**–**4**) decreased the inhibitory activity. The stilbenoid **19** and chalcone **20** also showed potent inhibition, consistent with previous reports [12,13,14]. The melanogenesis inhibitory effect of isolated compounds was also evaluated by measuring the melanin content in B16F10 melanoma cells. All the phenolic compounds except for compounds **3** and **20** reduced the melanin content at 10 μM without cytotoxicity (Figure 2B). Taken together, we can conclude that the phenolic compounds of *M. alba* leaves are active constituents with anti-melanogenesis activity. 

For maximum efficacy for the anti-melanogenesis effect, the extraction condition of *M. alba* leaves was optimized using response surface methodology. Our present study demonstrated the combinatorial effect of phenolic constituents of *M. alba* leaves on anti-melanogenesis activity, thus, tyrosinase inhibitory activity and phenolic content were selected as targets for optimization. Response surface analysis as well as statistical analysis showed that tyrosinase inhibition and total phenolic content were noticeably affected by the methanol concentration, followed by extraction temperature and extraction time. In addition, optimized extraction conditions were suggested for both maximum tyrosinase inhibition and total phenolic content, which was confirmed by experimental data. Taken together, our present study demonstrated that phenolic constituents are active constituents for the anti-melanogenesis activity of *M. alba* leaves. Our study also suggested the optimized extraction conditions of *M. alba* leaves as methanol concentration of 85.2%, temperature of 53.2 °C, and extraction time of 2.0 h, which gave 74.7% tyrosinase inhibition and 24.7 μg GAE/mg extract for maximum tyrosinase inhibition and total phenolic content. Conclusively, *M. alba* leaves are promising natural resources for development as cosmetics and food supplements and our study gives a strong support for their economic efficiency.

## 3. Experimental Section

### 3.1. General Information

NMR spectra were recorded on a DRX 500 MHz NMR spectrometer (Bruker, Karlsruhe, Germany). EI-mass spectra were obtained on a VG Autospec Ultima mass spectrometer (Waters, Milford, MA, USA). Semipreparative HPLC was performed using a Waters HPLC system (Waters) equipped with Waters 600 Q-pumps, a 996 photodiode array detector, and Waters Empower software using a Gemini-NX ODS-column (5 μm, 10 × 150 mm). Silica gel (70–230 mesh, Merck, Darmstadt, Germany) and Sephadex LH-20 (25–100 μm, Amersham Biosciences, Uppsala, Sweden) were used for open column chromatography (CC). Thin-layer chromatography (TLC) was performed on a precoated silica gel 60 F_254_ (0.25 mm, Merck). All other chemicals and reagents were analytical grade.

### 3.2. Isolation of Compounds ***1**–**20***

The leaves of *M. alba* (10.0 kg) were extracted twice with 80% MeOH (50 L, 24 h, room temperature) which yielded the methanolic extract (1.65 kg). The methanolic extract was then suspended in H_2_O and partitioned successively with *n*-hexane, CH_2_Cl_2_, EtOAc and *n*-BuOH (2 L each, twice, room temperatures). The CH_2_Cl_2_ fraction (109.2 g) was subjected to silica gel column chromatography with the mixture of CH_2_Cl_2_/MeOH to give seven fractions (M1–M7). M5 was subjected to column chromatography over Sephadex LH-20 eluting with MeOH to give five fractions (M5D1-M5D5). Compound **20** (2.5 mg) was obtained from M5D4 by semipreparative HPLC eluting with CH_3_CN/H_2_O. M4 was subjected to silica column chromatography with the mixture of *n*-hexane-EtOAc to give seven fractions (M4A-M4G). M4C was subjected to column chromatography over Sephadex LH-20 eluting with CH_2_Cl_2_/MeOH to give five fractions (M4C1-M4C5). Semipreparative HPLC of M4C4 eluting with CH_3_CN/H_2_O yielded compounds **13** (0.9 mg), **2** (0.2 mg), **3** (0.4 mg), **6** (0.6 mg), **7** (0.4 mg) and **8** (0.6 mg). M4B was subjected to column chromatography over Sephadex LH-20 eluting with CH_2_Cl_2_/MeOH to give seven fractions (M4B1-M4B7). M4D was subjected to column chromatography over Sephadex LH-20 eluting with CH_2_Cl_2_/MeOH to give four fractions (M4D1-M4D4). Compounds **4** (2.5 mg) and **5** (1.5 mg) was purified from M4D4 and M4B7, respectively, by semipreparative HPLC eluting with CH_3_CN/H_2_O. 

The EtOAc fraction (17.7 g) was subjected to silica column chromatography with the mixture of CH_2_Cl_2_/MeOH to give nine fractions (E1-E9). Compounds **9** (14.1 mg) was obtained from E3 by column chromatography over Sephadex LH-20 eluting with MeOH. Compound **19** (19.9 mg**)** was obtained from E7 by column chromatography over Sephadex LH-20 eluting with CH_2_Cl_2_/MeOH (1:1). E5 was subjected to RP-silica column chromatography with MeOH/H_2_O to give 6 fractions (E5A-E5F). E5B was subjected to column chromatography over Sephadex LH-20 eluting with MeOH to give 11 fractions (E5B1-E5B11). Compound **10** (13.0 mg) was obtained from E5B11 by recrystallization. Compounds **11** (2.6 mg), **14** (1.5 mg), and **15** (1.5 mg) were obtained from M5B6 by semipreparative HPLC eluting with CH_3_CN/H_2_O. E6 was subjected to column chromatography over Sephadex LH-20 eluting with MeOH to give eight fractions (E6A-E6H). E6F was subjected to column chromatography over Sephadex LH-20 eluting with CH_2_Cl_2_/MeOH to give six fractions (E6F1-E6F6). Compound **12** (5.0 mg) was obtained from E6F5 by recrystallization. Compound **1** (10.0 mg) was obtained from E6F6 by semipreparative HPLC eluting with MeOH/H_2_O. E8 was subjected to column chromatography over Sephadex LH-20 eluting with CH_2_Cl_2_/MeOH to give seven fractions (E8A-E8G). Column chromatography of E8D over Sephadex LH-20 eluting with MeOH yielded five fractions (E8D1-E8D5). Compounds **16** (0.5 mg) and **17** (0.5 mg) were obtained from E8D2 by semipreparative HPLC eluting with MeOH/H_2_O. Recrystallization of E9 yielded compound **18** (341.0 mg). 

### 3.3. Evaluation of Anti-Melanogenesis Activity

#### 3.3.1. Assessment of Tyrosinase Activity 

Tyrosinase inhibitory assays were performed using enzyme solution, which was prepared by the reconstitution of mushroom tyrosinase (Sigma, St. Louis, MO, USA) in 0.1 U/mL phosphate buffer (pH 6.5). Test sample was mixed with 50 μL enzyme buffer, and incubated for 5 min at 37 °C. Then, 50 μL tyrosine solution, which was diluted with phosphate buffer to 1 mM, was added and the enzyme reaction was allowed to proceed for 20 min at 37 °C. After incubation, the amount of dopachrome formed in the reaction mixture was determined by measuring the absorbance at 490 nm in an ELISA reader (Bio-Tek Synergy HT, Winooski, VT, USA). 

#### 3.3.2. Measurement of Melanin Contents

B16F10 mouse melanoma cells were obtained from the American Type Culture Collection (Manassas, VA, USA). Cells were cultured in Dulbecco’s modified Eagle’s medium (DMEM) supplemented with 10% fetal bovine serum (FBS), 100 IU/mL penicillin and 100 μg/mL streptomycin. Cells were maintained at 37 °C in a humidified atmosphere of 95% air-5% CO_2_. For the measurement of melanin content, B16F10 cells were stimulated with α-MSH and then treated with samples for 72 h. After washing with phosphate buffered saline (PBS), the cells were harvested and solubilized the melanin by vortexing in 1 N NaOH-10% DMSO at 80 °C. The melanin contents were measured by absorbance value at 490 nm with synthetic melanin as a standard. Cell viability was assessed by the 3-(4,5-dimethylthiazol-2-yl)-2.5-diphenyltetrazolium bromide (MTT) assay in an ELISA plate reader. 

## 4. Conclusions 

*M. alba* leaves exert anti-melanogenesis inhibitory activity as measured by tyrosinase inhibition and melanin content in B16F10 melanoma cells. Further fractionation of *M. alba* leaves resulted in the isolation of 20 phenolic compounds as active constituents. For maximum efficacy, optimized extraction conditions was derived using response surface methodology as methanol concentration of 85.2%, an extraction temperature, 53.2 °C, and an extraction time 2 h. Therefore, these results provide useful information about melanogenesis inhibitory constituents and optimized extraction conditions for *M. alba* leaves as potential cosmetic therapeutics to reduce skin hyperpigmentation.
